# CDC Grand Rounds: Getting Smart About Antibiotics

**DOI:** 10.15585/mmwr.mm6432a3

**Published:** 2015-08-21

**Authors:** Alicia Demirjian, Guillermo V. Sanchez, Jonathan A. Finkelstein, Shari M. Ling, Arjun Srinivasan, Lori A. Pollack, Lauri A. Hicks, John K. Iskander

**Affiliations:** 1 Epidemic Intelligence Service, CDC; 2 National Center for Immunizations and Respiratory Diseases, CDC; 3 Division of General Pediatrics, Boston Children’s Hospital; 4 Departments of Pediatrics and Population Medicine, Harvard Medical School, Boston, Massachusetts; 5 Centers for Medicare and Medicaid Services; 6 Division of Healthcare Quality Promotion, National Center for Emerging and Zoonotic Infectious Diseases, CDC; 7 Office of the Associate Director for Science, CDC

Each year in the United States, approximately two million persons become infected with antibiotic-resistant bacteria, at least 23,000 persons die as a direct result of these infections, and many more die from conditions complicated by a resistant infection (1). Antibiotic-resistant infections contribute to poor health outcomes, higher health care costs, and use of more toxic treatments (2). Although emerging resistance mechanisms are being identified and resistant infections are on the rise, new antibiotic development has slowed considerably (2).

Inappropriate antibiotic prescribing is an important and modifiable contributor to antibiotic resistance and is a problem in all health care settings (1). Inappropriate antibiotic use contributes to excess health care costs, promotes antibiotic resistance, and contributes to preventable adverse drug reactions. Antibiotics cause approximately 142,000 adult emergency department visits annually for adverse drug reactions; almost four out of five of these visits are for allergic reactions (3). Antibiotics also contribute to both health care- and community-associated *Clostridium difficile* infections, which are associated with considerable costs to patients and the health care system (1,4). In 2009, approximately $10.7 billion was spent on antibiotic therapy in the United States, including $6.5 billion, $3.6 billion, and $526.7 million in the outpatient, inpatient acute, and long-term care settings, respectively (5). The cost of antibiotic resistance to the U.S. economy is an estimated $20 billion annually in excess direct health care costs, with an additional $35 billion in lost productivity (1).

Antibiotic prescribing must be tracked to understand and improve antibiotic use. Several data sources and surveillance systems have been employed to examine antibiotic prescribing in hospitals and the community. These include the National Ambulatory Medical Care Survey, the National Hospital Ambulatory Medical Care Survey, the National Healthcare Safety Network, claims data from health plans and insurance companies, and data from private vendors (6). An accurate assessment of antibiotic prescribing, regardless of clinical setting, is important to identify opportunities to improve prescribing and maintain provider accountability.

An estimated half of antibiotic prescriptions given during pediatric ambulatory care visits are inappropriate, and over one quarter of adult prescriptions are for conditions for which antibiotics are rarely indicated (6,7). Health care providers prescribed 262.5 million courses of antibiotics in 2011 (842 prescriptions per 1000 persons), and prescriptions per 1,000 persons vary markedly according to geography (8). The highest prescribing states in 2011, Kentucky and West Virginia, had a rate more than twice that of the lowest prescribing state (Alaska). Why such variability exists is unclear, but this variability is unlikely to be explained by differences in population distribution and extent of infectious diseases.

Inappropriate antibiotic use is not limited to the outpatient setting. A recent evaluation of prescribing for inpatients in two specific scenarios (urinary tract infections in patients without indwelling catheters and treatment with intravenous vancomycin) identified that antibiotic use could have been improved in 37% of cases (9). Frequency of antibiotic prescribing among inpatients varies considerably among hospitals. A recent study of 19 hospitals that had completed data validation and submitted antibiotic use data from one or more patient care settings, found threefold differences in usage rates among 26 medical/surgical wards (9).

Visits for acute respiratory tract infections lead to more inappropriate antibiotic prescribing than visits for any other group of diagnoses. For example, antibiotic treatment for acute uncomplicated bronchitis is not recommended, and despite decades-long, widespread efforts to curb antibiotic prescribing, in 2010, 71% of all outpatient visits for this condition resulted in an antibiotic prescription (10). Similarly, overprescribing for pharyngitis is common. Only 5%–10% of pharyngitis cases among adults are caused by group A *Streptococcus*, for which antibiotic treatment is recommended, yet antibiotics are prescribed for approximately 60% of ambulatory care visits for adult pharyngitis (7). Outpatient antibiotic prescribing for children with acute respiratory tract infections has been decreasing since the mid- to late-1990s, but the rate of decline has slowed and might have reached a plateau (11). Several factors have been hypothesized to have contributed to this decrease, including the increased use of pneumococcal conjugate and influenza vaccines, national education campaigns to promote appropriate antibiotic use, and increasing concern among both the general public and health care professionals about antibiotic resistance.

In addition to the problem of overuse, antibiotic selection is often inappropriate. Prescribers often choose second- or third-line antibiotics, which are typically broad-spectrum drugs, despite established clinical practice guidelines recommending more targeted agents. Overuse of broad-spectrum antibiotics (e.g., second- or third-generation cephalosporins, fluoroquinolones) is especially problematic because of their potential for increased selection of resistant bacterial populations and their role in treating serious infections. Among U.S. ambulatory care visits during 2007–2009, broad-spectrum antibiotics accounted for 74% of antibiotics prescribed to patients during visits for respiratory conditions (7). Among hospitalized patients, 56% received an antibiotic during their stay and 30% received at least 1 dose of a broad-spectrum antibiotic (9).

## Improving Prescribing and Antibiotic Stewardship

The goal of antibiotic stewardship is to maximize the benefit of antibiotic therapy while minimizing harms to both the individual person and the community. Modest reductions in antibiotic prescribing can make a substantial impact. One study predicted that a 10% decrease in outpatient antibiotic prescribing rates would lead to a 16% decrease in *C. difficile* infection incidence in the community (12). Likewise, reducing exposure of hospitalized patients to broad-spectrum antibiotics by 30% can result in an estimated 26% reduction in inpatient *C. difficile* infections (9).

To reduce inappropriate prescribing, recent guidelines for common outpatient infections emphasize stringent case definitions and clinical observation for mild cases. For example, children aged ≥24 months with unilateral acute otitis media and mild symptoms are less likely to benefit from antibiotics, and are good candidates for close observation with shared decision-making that involves clinicians and caregivers. A mechanism for follow-up in 48–72 hours in such cases is recommended (8).

Several interventions have been shown to improve antibiotic prescribing. Audit and feedback involves tracking individual provider prescribing behaviors and giving feedback on their performance relative to peers or established benchmarks. Academic detailing is a method that adapts some strategies developed by pharmaceutical companies to influence prescribing behaviors that involves active, tailored, and personalized education to promote desired behaviors. Clinical decision support can be integrated with electronic health records to promote appropriate prescribing practices for common infections. Effective ambulatory care interventions have been summarized previously (13) and may be adapted to different settings. Although no single intervention can improve all prescribing behaviors in a given outpatient setting, multifaceted interventions involving active provider education appear to have the greatest benefit. Evidence increasingly supports the reduction of unnecessary antibiotic use through delayed prescribing strategies, where patients are given an antibiotic prescription to be filled within a specified timeframe if symptoms do not improve (8).

Measures promoting appropriate antibiotic prescribing in inpatient settings are primarily implemented through antimicrobial stewardship programs, which CDC recommends for all hospitals in the United States (http://www.cdc.gov/getsmart/healthcare/implementation/core-elements.html) (9). In a recent review of hospital interventions to improve antibiotic prescribing (14), both restrictive interventions (e.g., required approval from an infectious disease specialist to order certain antibiotics) and persuasive interventions (e.g., audit and feedback on prescribing behaviors or provider education) appeared to be equally effective after approximately 6 months. Interventions intended to reduce excess antibiotic prescribing have also been associated with reductions in *C. difficile* infection, and a meta-analysis of clinical outcomes found no significant increases in mortality caused by reductions in antibiotic prescribing when intervention groups were compared with controls (risk for mortality 0.92; 95% confidence interval = 0.81–1.06).

Educational campaigns aim to decrease inappropriate antibiotic prescribing by promoting judicious prescribing among providers and by increasing general public and provider knowledge about antibiotic resistance. Strategies to further employ appropriate antibiotic use messages include distribution of public health messages via pharmacies, child daycare centers, and workplaces. The CDC “Get Smart: Know When Antibiotics Work” and “Get Smart for Healthcare” campaigns (http://www.cdc.gov/getsmart) inform consumers and providers about antibiotic use and resistance, promote adherence to clinical practice guidelines, and support state- and local-level appropriate antibiotic use programs.

## Challenges, Success Factors, and Directions for the Future

Although guidelines exist for diagnosis and treatment of common infections, diagnostic uncertainty remains a challenge. Health care providers are frequently influenced by psychosocial factors which drive prescribing decisions, including concerns for both patient satisfaction with a clinical visit and potential negative consequences because of missed diagnoses (15). Providers are also concerned about losing dissatisfied patients to other providers who might be more likely to prescribe antibiotics. Patients who are aware of the potential risks for antibiotic overuse might still express a preference for antibiotic treatment because of perceived benefits. Antibiotic stewardship interventions and educational efforts aimed at addressing both diagnostic uncertainty and patient expectations will remain important.

Interventions to improve antibiotic prescribing have proven effective in the short-term and within specific settings. It remains less clear which interventions are sustainable and scalable. For this reason, strong stakeholder partnerships and buy-in at the personal, clinic, and health care system levels are fundamental to improving antibiotic prescribing. CDC is working with federal partners, including the Centers for Medicare and Medicaid Services, the U.S. Food and Drug Administration, and the Veterans Health Administration to improve prescribing. CDC partnerships with nonfederal stakeholders, such as vendors of antibiotic prescribing data, state health departments, and professional medical societies are also important.

In March 2015, *The National Action Plan for Combating Antibiotic-Resistant Bacteria* was released, outlining key actions to combat antibiotic resistance in the United States (https://www.whitehouse.gov). These actions include preventing the development and spread of resistant infections, increasing surveillance efforts, developing new drugs and diagnostic tests, and promoting international collaboration to prevent and control antibiotic resistance. In the United States, changes in health care delivery and increased implementation of quality measures provide opportunities to integrate antibiotic stewardship practices. Tracking antibiotic prescribing, regardless of clinical setting, is important in identifying opportunities to improve prescribing and maintain provider accountability. Priority should be placed on reducing prescribing for diagnoses for which inappropriate antibiotic prescribing is common (e.g., acute bronchitis) and on U.S. regions with higher antibiotic prescription rates. Reducing inappropriate antibiotic use and addressing the threat of antibiotic resistance is critical to improve health care quality and to safeguard patient safety across all health care settings.
